# Expectations of treatment outcomes in patients with spinal metastases; what do we tell our patients? A qualitative study

**DOI:** 10.1186/s12885-021-08993-0

**Published:** 2021-11-23

**Authors:** Anne L. Versteeg, Roxanne Gal, Raphaele Charest-Morin, Jorrit-Jan Verlaan, Hester Wessels, Charles G. Fisher, Helena M. Verkooijen

**Affiliations:** 1 Division of Imaging and Cancer, University Medical Center Utrecht, University of Utrecht, PO Box 85500, 3508 GA Utrecht, the Netherlands; 2 Division of Imaging and Cancer, Department of Radiotherapy, University Medical Center Utrecht, Universiteitsweg 100, 3584 CG Utrecht, the Netherlands; 3 Division of Spine, Department of Orthopaedics, University of British Columbia and Vancouver General Hospital, Vancouver, British Columbia Canada; 4 Department of Orthopaedic surgery, University Medical Center Utrecht, Utrecht, The Netherlands; 5 Department of Corporate Communications, University Medical Center Utrecht, Utrecht, The Netherlands

**Keywords:** Spinal metastases, Patient expectations, Health related quality of life, Patient physician communication, Qualitative research

## Abstract

**Background:**

Realistic pre-treatment expectations are important and have been associated with post-treatment health related quality of life (HRQOL). Patient expectations are greatly influenced by physicians, as they are the primary resource for information. This study aimed to explore the communication practices of physicians regarding treatment outcomes for patients with spinal metastases, and physician experiences with patients’ pre-treatment expectations.

**Methods:**

An international qualitative study using semi-structured interviews with physicians routinely involved in treating metastatic spine disease (spine surgeons, radiation and medical oncologists, and rehabilitation specialists) was conducted. Physicians were interviewed about the content and extent of information they provide to patients with spinal metastases regarding treatment options, risks and treatment outcomes. Interviews were transcribed verbatim and analyzed using a thematic coding network.

**Results:**

After 22 interviews data saturation occurred. The majority of the physicians indicated that they currently do not establish patients’ pre-treatment expectations, despite acknowledging the importance of these expectations. Spine surgeons often believe that patient expectations are disproportionate. Physicians expressed they manage expectations by detailing the most common risks and providing a broad but nonspecific overview of treatment outcomes. While the palliative intent seems clear to the physicians, their perception is that the implications of a palliative treatment remains elusive to most patients.

**Conclusion:**

This study highlights the current gap in patient-physician communication regarding expectations of treatment outcomes of patients with spinal metastases. These results warrant further research to improve communication practices and determine the effect of patient expectations on patient reported outcomes in this population.

**Supplementary Information:**

The online version contains supplementary material available at 10.1186/s12885-021-08993-0.

## Introduction

Management of patients with symptomatic spinal metastases is challenging and often involves a multidisciplinary approach including medical oncologists, radiation oncologists and spine surgeons. Treatment intent for patients with spinal metastases is almost exclusively palliative and is driven by the primary goal of improving or maintaining health related quality of life (HRQOL). The decision for a patient to accept a palliative cancer treatment is influenced by the expected effect of a treatment on their symptom burden and HRQOL [1]. In order to make an informed treatment decision, patients need to have realistic expectations regarding treatment outcomes, treatment risks, recovery time and their overall prognosis. Unrealistic pre-treatment expectations have been shown to result in decreased satisfaction with treatment outcomes, whereas realistic expectations have been associated with increased satisfaction [2–5]. A recent study demonstrated similar satisfaction rates after surgery and radiation therapy for the treatment of spinal metastases, despite significant differences in HRQOL outcomes between the two treatment groups. This may be explained by appropriately counseling patients towards realistic treatment expectations, however expectations were not evaluated by the authors [6].

In a recent systematic review on patient expectations regarding treatment outcomes of spinal surgery and advanced cancer care we demonstrated that patients tend to have overly optimistic expectations regarding symptom relief, recovery and prognosis [7]. Information provided by physicians was the primary resource for patients and important for patients’ understanding of their disease, treatment and outcomes of treatment [1, 7]. Expectations of treatment outcomes and subsequently satisfaction with treatment results are therefore greatly influenced by physicians [8]. Currently, little is known about the physician-patient communication practices in the field of spinal oncology.

The purpose of this qualitative study was therefore to explore how physicians communicate with patients with metastatic spine disease regarding treatment outcomes and examine their perception of patients’ pre-treatment expectations. In addition, we sought to explore the extent of the information provided by physicians regarding treatment options and risks, and patient prognosis. This study is the first of a series of studies from a phased project to determine patient expectations and the effect of patient expectations on patient reported outcomes.

## Methods

A qualitative study design using individual semi-structured interviews with thematic analysis was used. The consolidated criteria for reporting qualitative research (COREQ) were used [9]. The study was initiated in two tertiary spine centers in Canada and The Netherlands. The institutional review boards of the participating hospitals approved the research protocol.

### Participants

Health care providers were eligible to participate in the study if they had completed medical specialty training (no trainees) and were directly involved in the management of patients with spinal metastases. Due to the centralized spine oncology care, purposive sampling (criterion) was used [10]; i.e. participants who reflect the range of health care providers involved in the care of patients with spinal metastases in terms of treating physicians (spine surgeons and radiation oncologists), referring physicians (medical oncologists), physicians involved in the rehabilitation of patients with spinal metastases after treatment, and in terms of geographical location and time in practice were selected. Health care providers were approached for their participation in the study by the coordinating investigators in The Netherlands and Canada. Written informed consent was obtained from the participants. All participants were informed that they could opt out at any time.

### Interview methods

In person interviews were held at the hospitals of the participating health care provider. Interviews were conducted by researchers with experience in conducting qualitative interviews and with no prior relationship with the healthcare providers. A semi-structured interview guide containing broad and open questions was used to structure the interview to ensure consistency (Additional file 1). The interview guide was developed based on the input of physicians involved in the care of patients with spinal metastases and experienced qualitative researchers. The interview guide was finalized after performing pilot interviews. The following topics were discussed during the interviews; 1) information discussed with a patient about a treatment and treatment options, 2) information provided about expected treatment outcomes, and 3) physician perception of patient expectations. Participants were encouraged to describe their daily clinical experiences in their own language. When required, the interviewer could elicit additional information with open questions. The sample size of the current study was determined by data saturation [10]. Healthcare providers continued to be invited for interviews until no new information was retrieved on the topics addressed in the interview guide and no new topics were identified [10]. Data saturation occurred after 22 interviews were conducted.

### Data analysis

**Fig. 1:**
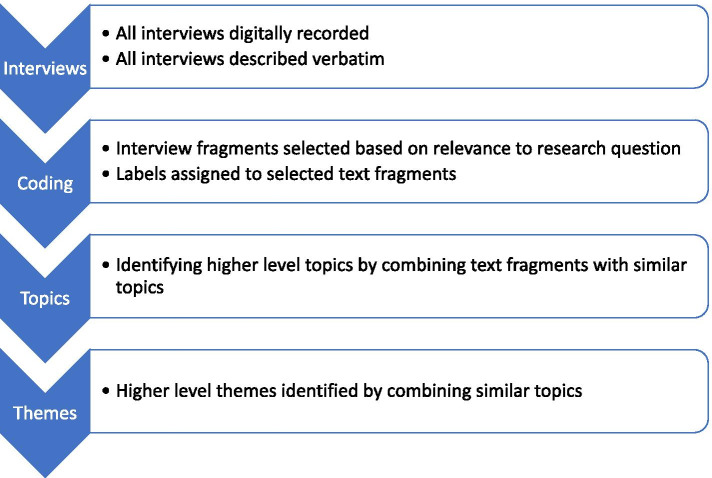
Flow diagram demonstrating steps in the qualitative analyses

All interviews were digitally recorded and verbatim transcribed by designated members of the research team. All identifiable information was removed from the interviews to ensure participant confidentiality. Interview data was processed using NVivo (Version 12; QSR International Pty Ltd., Melbourne, Australia, 2018). A primarily inductive thematic analysis according to Braun and Clarke was used to analyze the data [11]. Two reviewers (RG and AV), with training and experience in qualitative research methods, independently coded the data of the interviews. Coding was done by assigning labels to selected text fragments from the interviews that were relevant to the research question. Next, topics were identified by combining coded fragments with a similar topic. Lastly, topics were combined into overarching themes (Fig. 1). The coding structures of the themes and items of the researchers were compared. Consensus was reached through discussion in case of a discrepancy during the data analysis process. A senior qualitative researcher (HW) supervised the coding and analysis of the interview data.

## Results

Data saturation occurred after 22 interviews (The Netherlands, *N* = 14, Canada, *N* = 8). Between May and October 2019, interviews were conducted with spine surgeons (*N* = 11), radiation oncologists (*N* = 6), medical oncologists (*N* = 3) and rehabilitation specialists (*N* = 2). Interviews lasted between 18 and 53 min (median = 29.5 min). Characteristics of the health care providers are summarized in Table 1. No structural differences were identified based on different practice locations or context.

**Table 1:** Characteristics of participants

Characteristic	N
**Gender**	
• Female	• 10
• Male	• 12
**Specialty**	
• Spine surgery	• 11
• Radiation oncology	• 6
• Medical oncology	• 3
• Physiatry	• 2
**Fellowship training**	
• Yes	• 11
• No	• 11
**Interview time in minutes (median)**	29.5

The following themes and topics were identified during the analyses; patient counseling (theme) regarding treatment intent, prognosis, and risks and benefits (topics); treatment outcome expectations (theme) and the importance of expectations, managing expectations, perceptions of expectations and verifying expectations (topics); and improving patient counseling (theme).

### Counseling of a patient with spinal metastases

Physicians were asked to describe how they approach a consultation with patients with spinal metastases. The clinical environment of the first consultation for a patient with metastatic spinal disease varies across the specialties. The radiation oncologists and physiatrists indicated that they generally have a generous amount of time in the outpatient clinic for the first consultation. On the contrary, spine surgeons mentioned a limited allocated amount of time for a consultation in the outpatient clinic and that they are often facing emergency situations expediting the process before surgery.

*“With metastatic spine disease, unfortunately sometimes we don’t have the luxury of time and there is a lot of at least semi-urgent treatment that has to be initiated” [Spine surgeon 1]*

The majority of the radiation oncologists and spine surgeons indicated that during their consultation with a patient they state that the treatment is palliative with the objective to improve the patient’s health related quality of life. Although all physicians mentioned that most patients are aware that they have spinal metastases and that the treatment aim is palliative, yet they perceive that a substantial number of patients do not to fully understand the implications of a palliative treatment.

*“Some of them have the expectation that the metastasis will disappear, that the treatment will prolong their life” [Radiation oncologist 1]*

All surgeons and most of the radiation oncologists specified that they do not engage in discussing prognosis with patients as they find it challenging to predict and they deem it the responsibility of the referring medical oncologists. The medical oncologists mentioned that they actively inform patients about the palliative intent of any intervention for spinal metastases and explain the meaning of “palliative treatment”. The medical oncologists mentioned they will discuss life expectancy with the patient depending on patient preference; *“some patients want to know their prognosis while others prefer not” [Radiation Oncologist 1].* Medical oncologists acknowledge the discrepancy between the information given to the patient and their experience with the actual recollection of the patient.

All surgeons and radiation oncologists indicated that they discuss the most common treatment risks, mainly specific to their offered treatment (surgery or radiation), including wound infection, bleeding risk, neurological deterioration, hardware malposition/revision for the surgeons and nausea and gastro-intestinal problems for the radiation oncologists. The majority of the physicians noted that they counsel patients about treatment alternatives but, mostly limited to alternatives within their specialty field.

*“I do say something about the side effects, about fatigue, increase in pain, that kind of side effects of radiation but I don’t go into the dangerous territory of naming other things that I don’t have the expertise about. I think it is important to keep the expertise with the specialist” [Medical oncologist 1]*

Interestingly, only a few physicians mentioned to discuss the option of having no intervention done. Most physicians mentioned they are inclined to direct a patient towards a certain treatment but in the end, they believe that the final decision belongs to the patient.

### Expectations of treatment outcomes

Physicians were asked to what extent they discuss and establish treatment outcome expectations, and whether they think patients have realistic treatment outcome expectations. All physicians emphasized the importance of recognizing and managing patient expectations.

*“I think the better they know what they can expect, you are able to prevent disappointment” [Spine Surgeon 2]*

The majority of physicians indicated that unrealistic pre-treatment expectations may result in disappointment after treatment whereas realistic expectations may result in patients perceiving the treatment outcomes more positively. Furthermore, some physicians noted that by trying to bring the expectations too low, some patients will simply reject care.

*“I think it plays a big role. If you are expecting a desirable outcome and it doesn’t happen, you will be dissatisfied. So trying to align the expectations and experience, I think is important”[Spine surgeon 3]*

*“If you tell someone that this is a treatment that’s not likely to work, it may in fact be perceived that way and people give up hope and say, well forget it” [Spine surgeon 4]*

Few physicians indicated that although they believe that patient expectations are important, they perceived that these expectations have no influence on perceived treatment outcomes.

Despite recognizing the importance of patient expectations, medical oncologists, radiation oncologists and spine surgeons acknowledge that they mostly fail to actively ask patients about their expectations of treatment outcomes.

*“I don’t specifically ask patients regarding their expectations. I explain to them what they can expect, but I don’t actively ask patients them their expectations”[Spine surgeon 5]*

On the contrary, physiatrists actively engage in discussing patient’s expectations regarding recovery and other outcomes of treatment with the patient.

Most physicians explained that they manage patient expectations regarding outcomes by providing treatment information. The majority mentioned giving a broad yet nonspecific overview of treatment outcomes, and not tailored to the individual patient, in terms of expected pain reduction and for some physicians, changes in physical and/or neurological function after treatment. Physicians voluntarily remain vague with regards to recovery timelines as they feel it is highly variable. Physicians indicated that patients frequently have questions regarding logistical issues, treatment outcomes (pain and physical function) and length of the recovery period.

Although most physicians acknowledged not actively asking patients about their expectations, their perception of patient expectation varies. In general, surgeons believe that patients tend to have overly optimistic expectations regarding pain relief and underestimate the duration and magnitude of the recovery period. On the contrary, some physicians believe that given all the pre-treatment counselling patients have received, patients should have realistic expectations. Lastly, some health care providers noticed that some patients might fail to express their dissatisfaction after treatment.

*“Especially patients with instrumentation, they often have complaints about the hardware, or about the loss of mobility of their spine, or infections, or wound dehiscence. That is something that all patients underestimate” [Spine surgeon 6]*

*“The invasiveness of the surgery with regards to the muscular dissection and “bony” work results in significant amount of pain. Patients need to recover from that. I think the biggest thing patients underestimate is the recovery*” *[Spine surgeon 7]*

### Improving patient counseling

Physicians were asked about possible strategies to improve pre-treatment counselling for patients with spinal metastases. The majority of the physicians pointed out the lack of formal education resources available for these patients. Some physicians suggested involving a specialist nurse as a case manager within this patient population. Most physicians indicated that they would like to engage in regular spinal oncology multidisciplinary meetings. Physicians valued the multidisciplinary meetings as a great communication tool between the different specialists involved with the metastatic spine population. Only a few physicians mentioned already having regular multidisciplinary meetings. Yet, most physicians acknowledged the logistical difficulties with scheduling these meetings.

## Discussion

This qualitative study explored current communication practices by physicians to patients with spinal metastases regarding expected treatment outcomes, alternative treatments and the risks/benefits counseling. Furthermore, the perceptions of physicians of patient expectations regarding treatment outcomes were examined. A strategy used by most physicians to manage patient expectations is by providing information about the treatment in a generic way with a focus on pain reduction and emphasizing the risks associated with the treatment. Despite recognizing the upmost importance of treatment expectations, physicians often fail to actively ask patients regarding their specific outcome expectations. The majority of the surgeons expressed that they feel that patients overestimate treatment outcomes and underestimate the recovery period. These results suggest the current gaps in the patient-physician communication and open the discussion for improvement in the management of pre-treatment expectations of patients with metastatic spine disease.

Realistic expectations regarding life expectancy and treatment outcomes help patients make informed treatment decisions [7]. Previous studies have demonstrated that the majority of patients with advanced cancer overestimate their life expectancy and fail to understand that their disease is incurable [12–14]. A study of Enzinger et al. showed that only 17.6% of the patients with advanced cancer recalled that they were told about their estimated life expectancy by their medical oncologists [15]. Yet, the majority of these patients reported that they would have preferred to have their life expectancy disclosed. Patients who were disclosed their estimated life expectancy had a more accurate idea of their actual life expectancy compared to those who were unaware of their estimated life expectancy and furthermore, it did not affect negatively their emotional well-being or patient-physician relationship [15].

Weeks et al demonstrated that 69% of the patients with lung cancer and 81% of patients with colorectal cancer did not understand that palliative chemotherapy was not likely to cure their cancer [12]. In addition, the authors found an association between unrealistic expectations regarding the chance of cure and improved satisfaction with physician communication. This suggests that patients are less satisfied with physician communication when a more realistic but less positive view of the effects of palliative chemotherapy is communicated [12]. However, it is for patients crucial to understand that what the goals, risks and benefits of treatment are to be able to make an informed decision.

A review by Bernacki et al. highlighted the challenges associated with and importance of discussing goals of care in patients with serious illnesses including cancer [16]. Physicians perceive different barriers to address this topic including time constraints, the uncertainty regarding prognosis, ambiguity regarding who is responsible, the feeling of a lack of education to properly discuss goals of care and managing patient emotions [16]. Training of physicians in breaking bad news or to discuss end of life care has received considerable attention over the past two decades. However, training physicians to help patients understand that treatment is palliative has received less attention. Moreover, public websites about cancer care focused on curative treatment and often do not address palliative treatment [12]. In line with the review of Bernacki et al., the radiation oncologists and spine surgeons in our study revealed that they elicit not to discuss life expectancy with their patients as they deem it the responsibility of the medical oncologist. However, also the medical oncologists interviewed in our study reported not always engaging on a life expectancy conversation. This is in line with an article published by Daugherty et al. where only 43% of the medical oncologists regularly or always discuss life expectancy with their patients [17]. This might be explained by the fact that several studies have shown that life expectancy is difficult to predict and often overestimated by physicians [18]. Yet, whenever patients have unrealistic expectations regarding their life expectancy, physicians may not be able to adjust patients’ expectations regarding treatment options and outcomes of treatment. It is for patients crucial to understand that their HRQOL will be negatively affected by their cancer and treatments, yet the goal of treatment for the metastatic spinal disease is to maintain or improve their quality of life [19]. Unrealistic expectations may lead to patients accepting a treatment, including associated risks, that they might have refused had they had more realistic expectations regarding their life expectancy and post-treatment HRQOL.

In a recent systematic review, Witiw et al. investigated the relationship between pre-operative expectations and post-treatment satisfaction in patients who underwent elective spinal surgery [20]. In agreement with the statements made by the spine surgeons interviewed in our study, the authors found that patient expectations regarding treatment outcomes often are higher than the actual perceived outcomes. The authors concluded that a smaller gap between pre-treatment expectations and actual outcomes was associated with higher treatment satisfaction [20]. Interestingly, the authors found that for patients undergoing surgery for lumbar spinal stenosis there was a lack of an association between positive pre-treatment expectations and satisfaction with treatment outcomes. The authors suggested that this was due to characteristics of the specific patient population; it being an older population with multiple comorbidities and other age-related functional decline [20]. Predicting outcomes of an intervention in this patient population is harder which may lead to higher rates of unrealistically high expectations. These hypotheses may be translated to the complex population of patients with spinal metastases with often multiple comorbidities and lower functional status.

In a recent systematic review on patient expectations regarding treatment outcomes of spinal surgery and advanced cancer care we established that patient counseling is important for patients’ understanding of their disease process and treatment [7]. Yet, there is a discrepancy between patient and surgeon expectations regarding outcomes and the recovery process [21]. This may be explained by differences in understanding of the terms associated with spinal disease, treatment methods and the impact of a treatment on physical and mental well-being [21].

Multidisciplinary meetings or tumor boards are well established and have become the standard of care for patients with cancer [22]. Only a few of the interviewed physicians are currently participating in spinal oncology multidisciplinary meetings. This may be explained by the fact that until about a decade ago, the management of metastatic bone disease, including spinal metastatic disease, was not much of a concern as these patients were mostly at the end stage of their life [23]. With improved survival rates, more patients now require treatment for these skeletal-related events from spinal metastases such as pathological fractures and/or spinal cord compression [24]. A multidisciplinary approach has been shown to facilitate open communication, resulting in changes of treatment planning, improved treatment outcomes, and improved patient and physician satisfaction [22, 25]. In addition to the involved physicians from the various disciplines it should be considering to also include other health professionals such as advanced oncology nurse practitioners, physiotherapist and professionals from the mental health field. Considering the complexity of treating patients with spinal metastases and the increasing number of patients requiring treatment for spinal metastases, it may be advised to implement regular tumor boards specific to metastatic spine disease. Discussing patients in a multidisciplinary metastatic spine tumor board may also result in earlier referral of patients allowing for an appropriate amount of time to discuss treatment options, risks and outcomes, manage patient expectations and even decrease the extent of the surgical procedure.

A strength of this study is the qualitative study design as it allowed for an in-depth understanding of the communication practices of health care providers involved with metastatic spine patients. The study is further strengthened by the variety in background of the interviewees representing different medical specialties involved in the care of patients with spinal metastases, but also representing different geographical practice regions, hospital types and practice time. Yet, the number of medical oncologists interviewed in this study was low resulting in some imbalance in the representation of the specialties involved in the care of patients with spinal metastases. Despite covering different geographical regions, the study covered only two countries, which might limit the generalizability to other cultures and medical practices. A further limitation of the study was the absence of focus groups, which would have facilitated interdisciplinary discussion regarding communication practices.

## Conclusion

The majority of physicians participating in this study manage expectations of patients with spinal metastases by providing general information regarding treatment and treatment outcomes that is not specific to the patient. Generally, physicians fall short of asking patients regarding their treatment expectations, despite the fact that they acknowledge the importance of treatment expectations. Spine surgeons specifically noted that they experience patients having too high expectations of surgical outcomes. The results of this study suggest a current gap in patient-physician communication practices and can form the basis for future improvements, such as regular multidisciplinary clinics. Further studies are planned to determine patient expectations of treatments for spinal metastases and the impact of patient expectations on HRQOL and satisfaction.

## Supplementary Information

**Additional file 1.**

## Data Availability

The datasets generated during and/or analyzed during the current study are available from the corresponding author on reasonable request.
