# Supplementary material for: Expectations of treatment outcomes in patients with spinal metastases; what do we tell our patients? A qualitative study
Source: BMC Cancer. 2021 Nov 23;21:1263. doi: 10.1186/s12885-021-08993-0 (PMC8611925; doi:10.1186/s12885-021-08993-0)
Supplement: Supplementary file 1 — Additional file 1. [file 12885_2021_8993_MOESM1_ESM.pdf]

Interview guide

Patient expectations and perceptions in spinal oncology (PEPSO)

English version 1.5, February, 2019

Physician interview

Introduction

Thank you for agreeing to talk to us today. [*Short introduction of interviewer, name + job title]* Please let me know if you have any questions at this time.

Objective of the interview

We are interviewing patients who will receive treatment for a metastatic tumor of the spine to gain insight in their expectations regarding the outcome of treatment. In addition to patients, we will also interview relatives and caregivers of patients, as well as their physicians. The objective of this study is to improve communication between physicians and patients regarding treatment and outcomes of treatment.

Your opinion is important

We really appreciate your opinion and encourage you to give your point of view. There are no right or wrong answers.

Confidential

We will be taping this session and make notes based on the conversation in order to process the information carefully and to prevent the loss of any information. All the information gathered will be used for the purposes of this study only and your name will not be used in the summary reports.

The interview

The interview will last for about 45 to 60 minutes. If you feel that you would like to stop the interview, have a question or a comment, please let me know at any time.

Informed consent and questions

Do you have any questions at this time? We would like to sign the informed consent together with you.

Let's start with the interview.

Background information

Name: ______________________________ Date of interview: ______________
Age: ________ Gender: m / f

Specialty
- ☐ Orthopaedic surgeon
- ☐ Neurosurgeon
- ☐ Radiation oncologist
- ☐ Medical oncologist
- ☐ Other ____________________________

Hospital: ______________________________

Treatment options

What do you tell a patient when you discuss treatment options?

Gaining insight into the information that will be provided.

- What do you find important to discuss with a patient? Why?
- Do you discuss all the advantages and disadvantages of the different treatment options?
- Do you discuss the risks associated with treatment discussed?
- Do you inform patients about the palliative nature of the treatment?
- Which topics should be discussed more frequently in your opinion?
- Which factors influence what you do and do not discuss with a patient?

- What kind of questions do patients ask during a consult?
- How do you gain insight into the preferences and wishes of a patient?
- How do you use this information?
- What is the role of the patient in the treatment-decision making process?

- Are there other points regarding the conversation about treatment options that you would like to add?

Expectations – How is it going now?

To what extent do you ask about the patient's expectations regarding the outcomes of treatment during a consult?

Gaining insight into the extent to which physicians discuss expectations of treatment.

- Which factors influence the extent to which you verify their expectations?
- How do you get insight into the expectations regarding treatment outcomes?
- How do you use this information?

Do patients have realistic expectations?

Gaining insight into the experience of the physician of the expectations of patients regarding outcomes of their treatment.
Gaining insight into how physicians cope with realistic and unrealistic expectations.

- What do you do if you notice that a patient has unrealistic expectations?
- In your opinion, what is the effect of unrealistic pre-treatment expectations on patient recovery after treatment?
- And that of realistic expectations?
- What do you think patients need in order to create realistic expectations regarding their treatment outcomes?

Patients may have expectations regarding different domains (e.g. physical function, pain). What do you find important to discuss with a patient? Why?

- Which topics do you think, should be discussed more frequently?
- Which factors influence what you do and do not discuss with a patient?

- To what extent do you discuss with a patient what they can expect after their treatment regarding daily activities?
- To what extent do you discuss with a patient what they can expect after their treatment regarding the amount of assistance they need in daily life?
- To what extent do you discuss with a patient what they can expect after their treatment regarding their muscle strength?
- To what extent do you discuss with a patient what they can expect after their treatment regarding bowel and/or bladder control?
- To what extent do you discuss with a patient what they can expect after their treatment regarding pain in their back and/or neck?
- To what extent do you discuss with a patient what they can expect after their treatment regarding their mood?

- To what extent do you discuss with a patient what they can expect after their treatment regarding their personal relationships?
- To what extent do you discuss with a patient what they can expect after their treatment regarding their sexual life?

- Are there additional points that you would like to share regarding the expectations of patients?

Expectations – The future

Gaining insight into points for improvement prior to the treatment regarding patient expectations.

- Is there enough attention for the expectations of patients regarding treatment outcomes?
- Why is it (not) enough?
- What do you think can be improved prior treatment, for patients to be able to create realistic expectations?
- What is needed? There are no limitations.

- Are there additional points that you would like to share regarding the expectations of patients and how we can deal with this in the future?

Finishing up the interview

- Do you have any additional comments?

- Do you have any questions?

We would like to thank you for your time, if you don't have any further comments or questions, the interview has been completed. Please contact the study coordinator listed on the consent form should you have any additional comments or questions after this interview.
